# The spectrum of biopsy-proven glomerular diseases in a tertiary Hospital in Southern Brazil

**DOI:** 10.1186/s12882-021-02603-8

**Published:** 2021-12-13

**Authors:** Gustavo Gomes Thomé, Talissa Bianchini, Rafael Nazario Bringhenti, Pedro Guilherme Schaefer, Elvino José Guardão Barros, Francisco Veríssimo Veronese

**Affiliations:** 1 Graduate Program in Medicine: Medical Sciences, Federal University of Rio Grande do Sul, Porto Alegre, RS Brazil; 2 Division of Nephrology, Hospital de Clinicas de Porto Alegre, Federal University of Rio Grande do Sul, 2350, Ramiro Barcelos St, Porto Alegre, RS Brazil; 3 Division of Pathology, Hospital de Clinicas de Porto Alegre, Federal University of Rio Grande do Sul, Porto Alegre, RS Brazil

**Keywords:** Kidney biopsy registry, Primary glomerulonephritis, Secondary glomerulopathies, Clinical presentation, Nephrotic syndrome, Nephritic syndrome

## Abstract

**Background:**

The prevalence and distribution of glomerular diseases differ among countries, and the indication to perform a kidney biopsy varies among centres. In this study, we assessed the prevalence of primary and secondary glomerulopathies based on histological diagnoses, and the correlation between glomerulopathies and demographic and clinical data was evaluated.

**Methods:**

In this study, 1051 kidney biopsies were retrospectively reviewed between 2000 and 2018. Patient demographic, clinical and laboratory data were assessed. The prevalence of primary glomerulonephritis (PG) and secondary glomerulopathies (SG), as well as tubulointerstitial diseases (TIDs), hereditary nephropathies (HNs) and other diagnoses, were determined. The frequency of primary and secondary glomerulopathies was evaluated by age group, and the temporal variation in frequencies across three time periods (2000-2005, 2006-2011, and 2012-2018) was reported.

**Results:**

The prevalence of SG predominated (52.4%), followed by PG (29.6%), other diagnoses (10.7%), TID (6.6%) and HN (1.1%). Among the primary forms of glomerular disease, focal segmental glomerulosclerosis (FSGS) was the most common (37.3%), followed by IgA nephropathy (IgAN, 24.4%), membranous nephropathy (MN, 18.6%) and minimal change disease (MCD, 8.4%). Lupus nephritis (LN, 41.1%) was most common in patients with SG, followed by diabetic kidney disease (DKD, 17.8%), systemic vasculitis (SV, 10.2%) and secondary FSGS (2nd FSGS, 10%). Nephrotic syndrome was the most common clinical presentation in patients with PG and also in patients with DRD and 2nd FSGS, whereas in patients with IgAN and SV, nephritic syndrome was the main presentation. For the age group between 18 and 50 years, LN, FSGS and IgAN predominated; for patients aged between 51 and 65 years, the proportion of DKD and 2nd FSGS increased, and SV was more common in patients > 65 years. The temporal variation in PG across the three time periods showed a statistically significant increase in IgAN (*p* = 0.001) and a reduction in FSGS over time (*p* < 0.001). In SG, there was a reduction in LN (*p* = 0.027) and an increase in DKD (*p* < 0.001) over time, with a tendency for 2nd FSGS to decrease over time (*p* = 0.053).

**Conclusions:**

In the studied kidney biopsy registry, FSGS and IgAN were the most prevalent diagnoses in patients with PG, and LN and DKD were the most prevalent in patients with SG. Nephrotic syndrome was the major indication for biopsy. When comparing the temporal variation in glomerulopathies, there was a reduction in FSGS and an increase in IgAN in patients with PGs over time, and for patients with SGs, there was a reduction in LN with an increase in cases of DKD over time.

## Background

The prevalence of chronic kidney disease (CKD) has increased worldwide, and recent data show that glomerulopathies are the third leading cause of terminal chronic kidney disease in Brazil after arterial hypertension and diabetes mellitus [1]. As glomerular diseases have different clinical and laboratory manifestations, renal biopsy is a crucial approach for the diagnosis of glomerulopathy, allowing for appropriate therapeutic management and determination of patient prognosis based on the degree of activity and chronicity of glomerular and tubulointerstitial changes [2, 3].

The indications for renal biopsy and the distinct histopathological profiles in both primary and secondary glomerulopathies, as well as in genetic and tubulointerstitial diseases, have been described in recent studies [2, 4–7], and their prevalence has been found to vary over time. Optical microscopy and immunofluorescence analysis of tissues obtained by percutaneous renal biopsy are used to define an aetiological diagnosis; for some conditions, diagnoses are indicated based on clinical presentation and anatomopathological findings [2, 3]. However, there is an epidemiological discrepancy among different countries and continents due to the heterogeneity of the populations, ethnic differences, criteria for biopsy indication, environmental factors and socioeconomic restrictions specific to developing countries [2].

The prevalence of glomerulopathies varies worldwide. IgA nephropathy (IgAN) is the most common primary glomerulopathy in Asian countries [8, 9] and in some European countries [10, 11], while in Brazil [12–14] and the United States [4, 15, 16], focal segmental glomerulosclerosis (FSGS) is the prevalent glomerulopathy. A meta-analysis of the epidemiology of glomerulonephritis in Africa showed a higher prevalence of minimal change disease (MCD) and FSGS [17]. Regarding secondary glomerulopathies, studies have shown a higher prevalence of lupus nephritis (LN), diabetic kidney disease (DKD) and pauci-immune systemic vasculitis [8, 11, 18, 19]; however, the frequencies of these disorders have varied over the past two decades [6, 7, 15].

Temporal variation has been observed in the incidence rate of both primary and secondary glomerular diseases. Studies that evaluated the disease incidence over two or more decades showed an increasing trend in the prevalence of FSGS [7, 16, 20], MCD [8, 21], DKD [7, 15], pauci-immune systemic vasculitis [6, 7] and LN [6] as well as a temporal trend in the prevalence of IgAN [6, 9, 19, 20] and membranous nephropathy (MN) [8, 20]. Differences related to ethnicity have been reported, with IgAN [8, 18] being more prevalent in the Asian population, FSGS being more common in populations of African descent, and MN being more prevalent in the Caucasian population [16]. Additionally, it has been widely reported that the distribution of histological types varies based on age [6, 7, 18, 19].

Due to the heterogeneity of the Brazilian population in terms of geographic distribution, ethnic differences, temporal trends and age ranges, the prevalence and spectrum of glomerular diseases may differ around the country. In this study, we describe the histopathological diagnoses obtained via renal biopsy at our centre and analyse the correlation between primary and secondary glomerulopathies and the associated syndromes, clinical and laboratory characteristics, age groups and their prevalence over time.

## Methods

### Study design

This was a retrospective study that evaluated native kidney biopsies performed on patients aged 18 years or older between 2000 and 2018 at Hospital de Clínicas de Porto Alegre (HCPA), to determine the demographic profile, the form of clinical presentation and the histopathological diagnosis of renal disease. All data were obtained through the review of the patient medical records and were recorded anonymously. Patients underwent renal biopsy at the Nephrology Department or the Radiology Department of the HCPA during the period described. Patients who underwent biopsy at the Radiology Department were located through a query processed by the computer technology system of the HCPA.

The clinical, laboratory and histopathological data for all patients were registered in a database, and the histology data were obtained from the Renal Biopsy Registry of the Nephrology Service and the patient medical record. All methods were carried out in accordance with the relevant guidelines and regulations of the HCPA. This study was approved by the Research Ethics Committee of the Federal University of Rio Grande do Sul (CEP/UFRGS) under the number 3.159.116. The informed consent form was waived by the Research Ethics Committee and the Institutional Review Board of the Federal University of Rio Grande do Sul due to the retrospective nature of the study, and a confidentiality agreement was signed by the research team with access to patient medical records.

### Clinical and laboratory data

The clinical variables analysed included name, age at the time of biopsy, age group (18-35, 36-50, 51-65 and > 65 years), sex, ethnicity, date of biopsy, weight, and presence of systemic arterial hypertension (SAH) and diabetes mellitus (DM). The clinical presentation syndromes were searched in the medical records based on the clinical and laboratory data of the patients at the time of the biopsy and were defined as eight categories: 1) non-nephrotic proteinuria (< 3.5 g/day) with normal renal function; 2) isolated haematuria (≥3 red blood cells per high-power field in qualitative urine examination, with normal renal function and without proteinuria); 3) proteinuria and haematuria together, with normal renal function; 4) nephrotic syndrome (proteinuria > 3.5 g/day, serum albumin < 3.0 g/dL, oedema, with or without dyslipidaemia); 5) nephritic syndrome (non-nephrotic proteinuria < 3.5 g/day, haematuria, arterial hypertension, with or without loss of renal function); 6) nephrotic and nephritic syndrome together (proteinuria > 3.5 g/day with haematuria and loss of renal function); 7) proteinuria with loss of renal function (serum creatinine > 1.20 mg/dL for men and > 1.10 mg/dL for women); and 8) isolated loss of renal function, without proteinuria or haematuria.

Laboratory variables were obtained from the exams closest to the date of the renal biopsy no more than 3 months before or after the procedure. The variables collected included the following: serum creatinine levels; estimated glomerular filtration rate (eGFR) evaluated using the Modification of Diet in Renal Disease (MDRD) formula until 2009, and after 2009 using the Chronic Kidney Disease-Epidemiology Collaboration (CKD-EPI) formula; the 24-h proteinuria or proteinuria-creatininuria index (IPC) in the urine sample; the presence of haematuria in the urinary sediment; serum albumin levels; total cholesterol levels; HDL cholesterol levels; LDL cholesterol levels; and triglyceride levels. The serological variables evaluated were complement C3, complement C4, anti-nuclear factor (ANA), anti-DNAds, anti-neutrophil cytoplasmic antibody (ANCA), anti-glomerular basement membrane antibody (anti-MBG), anti-HIV, anti-HCV, HBsAg, cryoglobulins and serum protein immunoelectrophoresis.

### Histological classification

Histological findings were divided into the following four categories: primary glomerulonephritis, secondary glomerulopathies, hereditary nephropathies and tubulointerstitial diseases. Primary glomerulonephritis included minimal change disease, focal segmental glomerulosclerosis, membranoproliferative glomerulonephritis (MPGN), IgA nephropathy, membranous nephropathy, and mesangial proliferative glomerulonephritis (MesPGN). Secondary glomerulopathies were categorized as lupus nephritis; diabetic kidney disease; systemic vasculitis included ANCA-associated vasculitis, anti-glomerular basement membrane disease and cocaine-associated vasculitis; secondary FSGS (2nd FSGS) included HIV-associated nephropathy (HIVAN), obesity-associated glomerulopathy, drug-induced glomerulopathies, and other pathologies manifested by 2nd FSGS; secondary membranoproliferative glomerulonephritis (2nd MPGN) included hepatitis C virus associated glomerulonephritis, cryoglobulinemic vasculitis, and C3 glomerulopathy (C3 glomerulonephritis, C3GN and Dense Deposit Disease, DDD); thrombotic microangiopathy (TMA); all renal disease related to monoclonal gammopathies were described together as A-MM-LCDD (amyloidosis, multiple myeloma and light chain deposition disease); and other glomerulopathies included post-infectious glomerulonephritis, secondary membranous nephropathy, HIV-associated nephropathy other than FSGS, Henoch-Schönlein purpura, Sjögren’s syndrome and hypertensive nephrosclerosis. Renal biopsies that did not fall into the categories described above were classified as other diagnoses and included normal kidney, terminal kidney, biopsy without a conclusive diagnosis, or non-representative biopsy.

Tubulointerstitial diseases included acute tubulointerstitial nephritis (ATIN), chronic tubulointerstitial nephritis (CTIN) and acute tubular necrosis (ATN). Hereditary diseases included thin basement membrane disease (TBMD), Alport’s syndrome and Fabry’s disease.

We only included one diagnosis per patient. In patients with more than one histological finding, only the disease that was more relevant clinically and histologically was considered.

### Percutaneous renal biopsy

Renal biopsies were guided in real-time by ultrasound. Biopsies were performed by the Nephrology Service with a 16G needle and by the Radiology Service with an 18G needle. The histological sections were placed in buffered formalin for light microscopy and stained with haematoxylin and eosin (HE), Schiff’s periodic acid (PAS), periodic Schiff-methenamine (PASM) and Masson’s trichrome (TM). The frozen fragment in liquid nitrogen was evaluated by immunofluorescence using antibodies against immunoglobulins IgG, IgA, and IgM, C3 and C1q, Kappa and Lambda light chains, and fibrinogen. For selected cases, according to clinical indication, electronic microscopy (EM) was performed. The decision to include EM was made when optical microscopy and immunofluorescence did not provide a histological diagnosis or when hereditary nephropathy was suspected.

The total number of glomeruli on optical microscopy and immunofluorescence were evaluated, and the percentage of interstitial fibrosis and tubular atrophy (IFTA) was quantified as no fibrosis or fibrosis in < 25%, 25-50% or > 50% of the sample. Biopsies in which the diagnosis was not established due to insufficient or inadequate histological material were considered non-representative.

### Statistical analysis

The data are presented as absolute frequency and percentage, mean ± standard deviation (SD), or median and interquartile range. The association between categorical variables was analysed using a chi-square test or Fisher’s exact test. The Kolmogorov-Smirnov and Shapiro-Wilks tests were employed to determine the normality of the variables. An independent t-test or ANOVA was used for normally distributed variables, and the Mann-Whitney or Kruskal-Wallis test was used for non-normally distributed variables. The distribution of primary and secondary glomerulopathies by age group and their prevalence in the three observation periods (2000-2005, 2006-2011 and 2012-2018) were analysed using a chi-square test. All analyses were performed using SPSS software (version 21.0, SPSS Inc., Chicago, IL). A *P* value less than 0.05 was considered statistically significant.

## Results

A total of 1051 renal biopsies were performed from January 2000 to December 2018. The demographic and clinical data of the patients are shown in Table 1. The distribution was similar between sexes, and there was a predominance of Caucasians, representing 85.5% of the cases. The most frequent presentation syndrome was nephrotic syndrome, followed by proteinuria, proteinuria and haematuria and nephritic syndrome. SAH was present in 83.3% of the 706 patients who had this information available, and DM was present in 26.3% of 693 patients. The number of biopsies performed by the Nephrology department (51.2%) and the Radiology department (48.8%) was similar, and only 2.8% of the biopsies were non-representative. Most biopsies had mild fibrosis, with IFTA < 25% in 44.7% of biopsies, IFTA 25-50% in 30.3% of biopsies, representing moderate fibrosis, and IFTA > 50% in 25% of biopsies, indicating severe fibrosis.

**Table 1:** Clinical and demographic data

	N(%)
Total	**1051**
Age	44.9 ± 16.1^a^
Sex (M/F)	
Male	495(47.1)
Female	556(52.9)
Ethnicity	
Caucasian	899(85.5)
Afro-descendant	148(14.9)
Amerindian	4.0(0.4)
Clinical syndromes^b^	
proteinuria	205(21.5)
haematuria	10(1)
proteinuria and haematuria	194(20.4)
nephrotic syndrome	366(38.4)
nephritic syndrome	160(16.8)
nephritic and nephrotic syndrome	18(1.9)
Loss of renal function^c^	616(63.7)
Systemic arterial hypertension^d^	588(83.3)
Diabetes mellitus^e^	182(26.3)
Renal biopsy by executor	
Nephrology department	538(51.2)
Radiology department	513(48.8)
Total number of glomeruli on OM	15 ± 9.5
IFTA percentage	
Without fibrosis	132(14.3)
< 25%	413(44.7)
25-50%	280(30.3)
> 50%	98(10.6)
Non-representative biopsies	29(2.8)

*OM* optical microscopy, *IFTA* interstitial fibrosis and tubular atrophy

^a^mean ± standard deviation

^b^*N* = 953

^c^*N* = 967

^d^*N* = 706

^e^*N* = 693

**Fig. 1:**
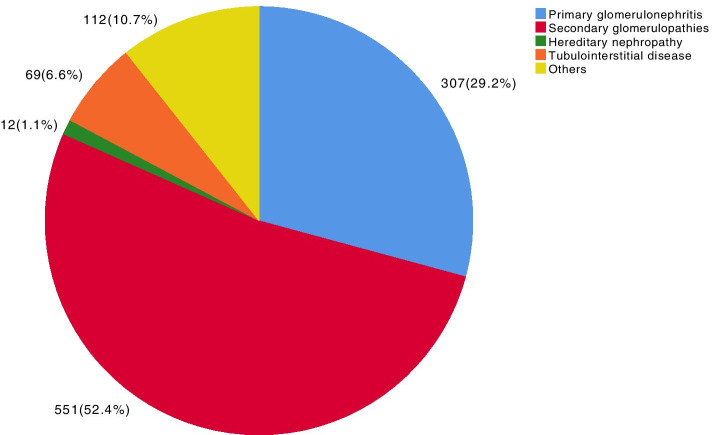
Presentation of the percentage of primary and secondary glomerulopathies, hereditary diseases, tubulointerstitial diseases and other diagnoses based on 1051 kidney biopsies performed between 2000 and 2018. Tubulointerstitial diseases: acute tubulointerstitial nephritis, chronic tubulointerstitial nephritis, and acute tubular necrosis. Hereditary diseases: Alport syndrome, Fabry disease, and thin basement membrane disease. Other diagnoses: normal kidney, terminal kidney, biopsy without diagnosis, and non-representative biopsy

The distribution of the histological findings in the 1051 biopsies is presented in Fig. 1. Secondary glomerulopathies were predominant, followed by primary glomerulopathies, tubulointerstitial diseases, hereditary nephropathies, and other diagnoses, including biopsies with normal kidney, terminal kidney, biopsy without a conclusive diagnosis, non-representative biopsy, or diagnoses that did not fit into the previous categories. Of the 24 patients diagnosed with hypertensive nephrosclerosis, 7 patients had secondary glomerulopathies with 2nd FSGS detected in addition to arterionephrosclerosis; the remaining 17 patients were excluded because the anatomopathological study showed only arterionephrosclerosis with medial thickening of the vascular wall, arteriolar hyaline deposits, and intimal fibrosis.

**Fig. 2:**
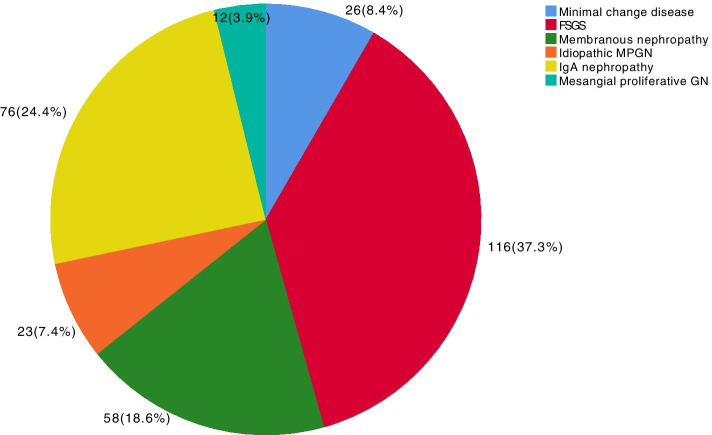
Frequency of primary glomerular diseases (*N* = 311). FSGS: focal segmental glomerulosclerosis, MPGN: membranoproliferative glomerulonephritis, GN: glomerulonephritis, mesangial proliferative GN: mesangial proliferative glomerulonephritis

**Fig. 3:**
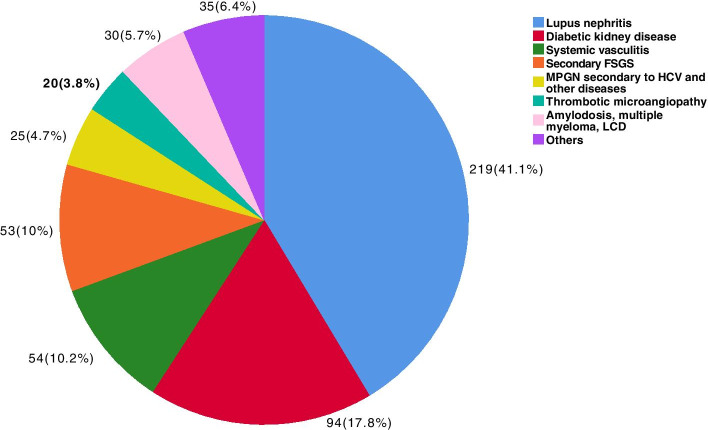
Frequency of secondary glomerular diseases (*N* = 530). FSGS: focal segmental glomerulosclerosis, MPGN: membranoproliferative glomerulonephritis, HCV: hepatitis C virus, LCDD: light chain deposition disease, Systemic vasculitis: ANCA-associated vasculitis, cocaine/levamisole associated vasculitis, and anti-glomerular basement membrane disease. Secondary FSGS: HIV-associated nephropathy (HIVAN), obesity-related glomerulopathy, and other secondary FSGS. MPGN and other diseases: hepatitis C virus associated glomerulonephritis, and cryoglobulinemia vasculitis. Other diagnoses: postinfectious glomerulonephritis, secondary membranous nephropathy, HIV-associated nephropathy not related to FSGS, Henoch-Schönlein purpura, Sjögren’s syndrome, and hypertensive nephrosclerosis

**Fig. 4:**
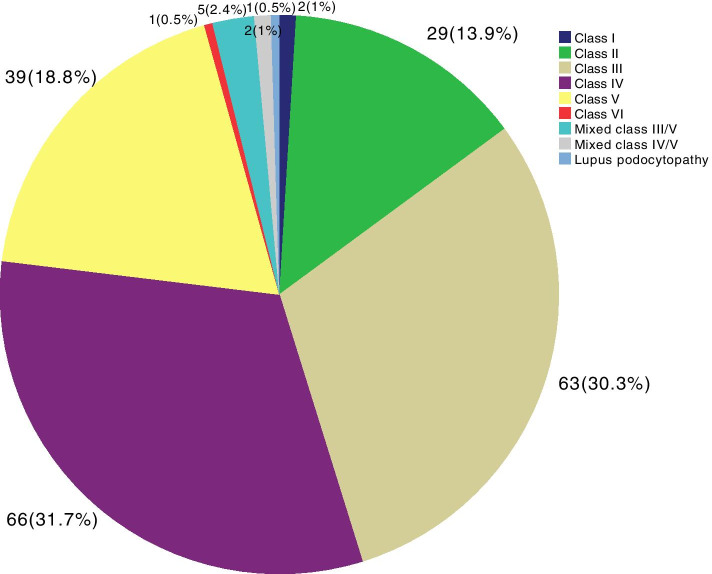
Frequency of the classes of lupus nephritis (*N* = 219)

**Fig. 5:**
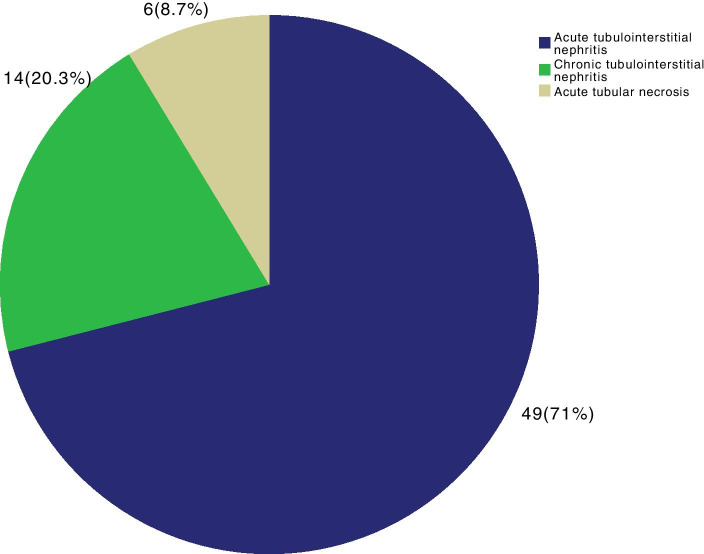
Frequency of tubulointerstitial renal disease (*N* = 69)

Among the patients with primary glomerulopathies (Fig. 2), the most frequent diagnosis was FSGS, followed by IgAN, MN, MCD, MPGN and MesPGN. Among the patients with secondary glomerulopathies (Fig. 3), LN was the most common diagnosis, followed by DKD, systemic vasculitis, 2nd FSGS, A-MM-LCDD, 2nd MPGN, and TMA. Other causes of secondary glomerulopathy accounted for 6.4% of the patients. The frequencies of each LN histological class are presented in Fig. 4, showing a predominance of diffuse and focal proliferative lupus nephritis, followed by membranous nephropathy. Among tubulointerstitial diseases (Fig. 5), ATN was prevalent, followed by CTIN and ATIN.

**Fig. 6:**
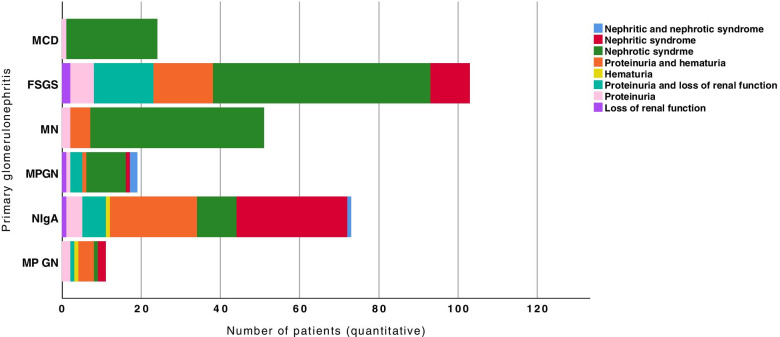
Distribution of the presentation of clinical syndromes according to each primary glomerulonephritis. *P* < 0.001 (Fisher’s exact test, chi-square test); the proportion of some of the primary glomerulonephritis patients differed significantly from each other based on the clinical presentation (*p* < 0.05). MCD: minimal change disease; FSGS: focal segmental glomerulosclerosis; MN: membranous nephropathy; MPGN: membranoproliferative glomerulonephritis; IgAN: IgA nephropathy; MesPGN: mesangial proliferative glomerulonephritis

Figure 6 shows the distribution of primary glomerulopathies according to the clinical syndrome. The percentage value in each glomerular pathology category described below represents the number of patients with this specific clinical manifestation. Nephrotic syndrome was prevalent in MCD (95.8%) and MN (86.3%) and in approximately half of the patients with FSGS (53.4%) and MPGN (52.6%). Presentation by nephritic syndrome was more associated with IgAN (38.4%). Isolated haematuria was uncommon and was identified only in patients with MesPGN (9.1%) and IgAN (1.4%). Proteinuria with haematuria predominated in patients with MPGN (36.4%) and IgAN (30.1%).

**Fig. 7:**
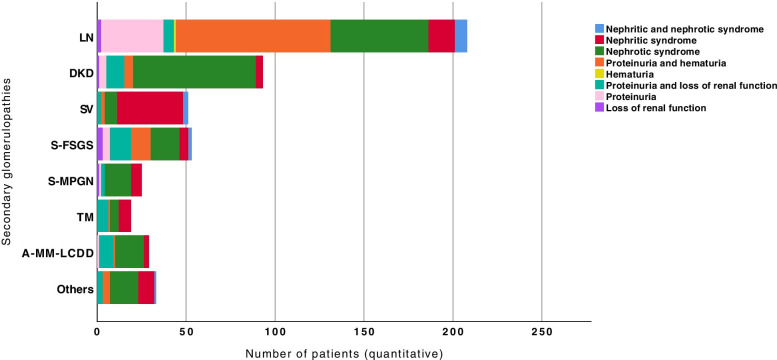
Distribution of the presentation of clinical syndromes according to each secondary glomerulopathy. *P* < 0.001 (Fisher’s exact test, chi-square test); the proportion of some of the secondary glomerulopathies patients differed significantly from each other based on the clinical presentation (*p* < 0.05). LN: lupus nephritis; DKD: diabetic kidney disease; SV: systemic vasculitis; S-FSGS: secondary focal segmental glomerulosclerosis; S-MPGN: secondary membranoproliferative glomerulonephritis; TMA: thrombotic microangiopathy; A-M-LCDD: amyloidosis, multiple myeloma, light chain deposition disease

Fig. 7 shows the distribution of secondary glomerulopathies. Among the LN patients, 37% had nephrotic syndrome, nephritic syndrome, or a mixture of nephrotic/nephritic syndrome as the clinical and laboratory manifestation, and in 41.8% of patients, the presentation was a combination of proteinuria and haematuria. Nephrotic syndrome was prevalent in patients with DKD (74.2%), 2nd MPGN (60%) and A-MM-LCDD (55.2%), and nephritic syndrome was observed in 72.5% of the patients with systemic vasculitis, 36.8% of those with TMA and 27.3% of those with other secondary glomerulopathies. Proteinuria with loss of renal function occurred in one-third of patients with TMA (31.6%), A-MM-LCDD (27.6%) and 2nd FSGS (22.6%). Loss of renal function as the only clinical manifestation was infrequent and observed in only 5.7% of patients with 2nd FSGS and in 4% of patients with 2nd MPGN.

The demographic, clinical and laboratory data of the patients with primary glomerulopathies are shown in Table 2. In the analysis based on the four age groups, MCD predominated in patients aged 18-35 years, while FSGS, MPGN, IgAN and MesPGN were more prevalent in the 18-35- and 36-50-year age groups. For the 36-50- and 51-65-year age groups, MN was the most frequent glomerulopathy. There was a higher prevalence of women among the cases of MCD and of men among the cases of FSGS and IgAN, with a similar distribution observed for both sexes in the other pathologies. The white race predominated in all primary glomerulopathies (> 90%), but in MCD, 19.2% of patients were of African descent. Nephrotic proteinuria and hypoalbuminemia were the most common clinical presentations in patients with MCD, FSGS, MN and MPGN, while in patients with IgAN and MesPGN, the prevalence of subnephrotic proteinuria was more frequent. In patients with primary glomerulonephritis, changes in serological markers were found with serum C3 levels decreased in patients with MPGN (*p* < 0.001) and HBsAg levels decreased in those with IgAN and MesPGN (*p* = 0.022). The other serological markers were not significantly different (*p* > 0.05) among all of the primary diseases.

**Table 2:** Demographic, clinical and laboratory parameters in primary glomerulonephritis

	MCD	FSGS	MN	MPGN	IgAN	MesPGN	*P* value
Age (years, %)							0.012^1^
18-35	57.7	40.5	20.7	30.4	51.3	50	
36-50	19.2	27.6	24.1	26.1	26.3	33.3	
51-65	19.2	25	37.9	21.6	17.1	8.3	
> 65	3.8	6.9	17.2	21.8	5.3	8.3	
Sex (%)							0.03
Female	73.1	39.7	50	56.5	31.6	58.3	
Male	26.9	60.3	50	43.5	68.4	41.7	
Ethnicity (%)							0.647
Caucasian	80.8	89.7	91.4	95.7	93.4	91.7	
Afro-descendant	19.2	9.5	8.6	4.3	5.3	8.3	
Amerindian	0	0.9	0	0	1.3	0	
DM (%)	10	9.7	14.7	6.7	11.3	0	0.960
SAH (%)	63.6	84.2	94.1	93.3	81.5	83.3	0.173
Weight	72 ± 12	73 ± 15	73 ± 19	79 ± 10	78 ± 15	65 ± 10	0.395^2^
Serum creatinine	0.8 (0.5-1.5)	1.5 (1.0-2.0)	0.9 (0.8-1.3)	2.0 (1.2-3.1)	1.7 (1.1-2.2)	1,1 (0.7-2.3)	< 0,001^3^
eGFR	101 (46-124)	56 (34-90)	72 (46-103)	38 (18-60)	47 (31-81)	66 (37-109)	0.001
Proteinuria	7.7 (5.6-8.8)	4.6 (2.3-7.3)	7.0 (4.3-9.9)	7.8 (2.4-9.7)	1.9 (1.1-3.7)	1.6 (0.7-2.6)	< 0.001
Serum albumin	2.1 ± 0.9	2.9 ± 0.9	2.5 ± 0.7	2.5 ± 0.7	3.8 ± 0.7	3.8 ± 0.6	< 0.001
Total Cholesterol	421 (321-522)	257 (187-392)	282 (199-344)	236 (194-317)	198 (155-233)	180 (160-225)	< 0.001
Triglycerides	221 (179-279)	179 (117-277)	194 (134-284)	235 (139-339)	130 (99-219)	176 (80-191)	0.006
C3^a^	143 ± 34.5	126 ± 31.3	134 ± 27.4	99 ± 43.3	114 ± 29	114 ± 33.3	< 0.001
C4^b^	38.8 ± 13.9	34.5 ± 12.5	34.8 ± 14.6	29.2 ± 20.2	30.5 ± 11.7	28 ± 12	0.266
Anti-HIV (N,%)^c^	1(6.7)	0	1(2.6)	0	1(1.9)	0	0.413
Anti-HCV (N,%)^d^	1(6.7)	1(1,2)	1(2,6)	0	4(7,3)	0	0.370
HbsAg (N,%)^e^	0	0	0	0	4(7.3)	1(12.5)	0.022

*DM* diabetes mellitus, *SAH* systemic arterial hypertension, *eGFR* estimated glomerular filtration rate, *MCD* minimal change disease, *FSGS* focal segmental glomerulosclerosis, *MN* membranous nephropathy, *MPGN* membranoproliferative glomerulonephritis, *IgAN* IgA nephropathy, *MesPGN* mesangial proliferative glomerulonephritis

^1^N(%): Chi-square test

^2^Mean ± standard deviation: ANOVA

^3^Median (interquartile range): Kruskal-Wallis test

Number of patients with measured serological parameters: ^a^C3: 197; ^b^C4: 197; ^c^anti-HIV: 214; ^d^anti-HCV: 216; ^e^HbsAg: 214

Among the patients with secondary glomerulopathies (Table 3), LN predominated in patients under 35 years old, DKD in patients 36-50 years of age, and systemic vasculitis in those over 50 years. Secondary FSGS had a higher prevalence in patients between 36 and 65 years of age, 2nd MPGN in those between 51 and 65 years of age, and A-MM-LCDD in those above 50 years of age. Lupus nephritis and TMA predominated in women, and 2nd MPGN and other secondary glomerulopathies predominated in men. Secondary forms prevailed in Caucasian patients, but for those of African descent, there was a higher frequency of DKD, 2nd FSGS, A-MM-LCDD and other pathologies. Laboratory and serological exams showed significant differences according to the data presented in Table 3, where ANA (*p* < 0.001; *N* = 533) and anti-DNAds (*p* = 0.035; *N* = 450) were prevalent in patients with LN, and ANCA titers (*p* = 0.011; *N* = 305) prevailed in patients with systemic vasculitis (data not shown in Table 3).

**Table 3:** Demographic, clinical and laboratory parameters in secondary glomerulopathies

	LN	DKD	SV	S-FSGS	S-MPGN	TMA	A-MM-LCDD	Others	*P* value
Age (years; %)									< 0.001^1^
18-35	58.9	10.6	18.5	18.9	24	45	10	26.5	
36-50	26.5	21.3	18.5	37.7	24	40	13.3	17.6	
51-65	11.9	57.4	33.3	34	48	5	40	44.1	
> 65	2.7	10.6	29.6	9.4	4	10	36.7	11.8	
Sex (%)									< 0.001
Female	85.4	43.6	51.9	43.4	36	75	60	32.4	
Male	14.6	56.4	48.1	56.6	64	25	40	67.6	
Ethnicity (%)									0.007
Caucasian	84	76.6	94.4	75.5	84	95	76.7	70.6	
Afro-descendant	16	23.4	5.6	22.6	12	5	23.3	29.4	
Amerindian	0	0	0	1,9	4	0	0	0	
DM (%)	8.9	100	22.9	28.6	15.8	9.1	5.9	25	< 0.001
SAH (%)	71.4	95.4	80.6	90.5	78.9	81.8	94.1	96	< 0.001
Weight	68 ± 16	78 ± 17	74 ± 18	72 ± 15	73 ± 19	87 ± 31	64 ± 13	81 ± 14	0.007^2^
Serum creatinine	0.8(0.6-1.1)	2.8 (1.4-4.5)	3.1(2.2-5.5)	2.0 (1.2-3.5)	1.8 (1.4-3.2)	4.1(3.1-6.6)	2.9 (0.9-4.9)	1.5 (1.0-2.3)	< 0.001^3^
eGFR	98 (62-120)	22 (13-50)	19 (9.9-25)	32 (16-53)	37 (18-48)	13 (8.3-17)	21 (11-69)	47 (24-82)	< 0.001
Proteinuria	2.1(0,7-4.2)	6.0 (3.7-9,2)	1.5 (0.9-3.2)	2.5 (1.5-6.5)	5.0 (2.9-6.7)	1.9 (1.4-3.0)	4.8 (2.5-8.6)	3.5 (1.1-6.2)	< 0.001
Serum albumin	3.0 ± 0.7	2.8 ± 0.6	3.2 ± 0.6	3.2 ± 1.0	2.5 ± 0.8	3.2 ± 0.6	2.9 ± 1.0	2.9 ± 0.8	0.009
Total Cholesterol	229 (176-284)	197 (158-239)	217 (150-255)	208 (172-288)	199 (123-284)	171 (157-227)	220 (181-261)	242 (186-287)	0.081
Triglycerides	170 (114-255)	170 (121-270)	159 (108-165)	250 (172-418)	170 (129-257)	219 (116-375)	170 (127-203)	127 (107-274)	0.011
C3^a^	72 ± 33	125 ± 33	121 ± 31	120 ± 40	92 ± 41	110 ± 34	119 ± 31	108 ± 47	< 0.001
C4^b^	12 ± 11	35 ± 13	29 ± 13	30 ± 13	21 ± 12	30 ± 11	41 ± 20	27 ± 18	< 0.001
Anti-HIV (N,%)^c^	2 (1.2)	11 (12.4)	0	30(58.8)	6 (24)	1 (6.7)	0	8(24.2)	< 0.001
Anti-HCV (N,%)^d^	3 (1.7)	26 (29.5)	4 (7.8)	9 (18)	18 (75)	2 (11.8)	1 (3.7)	10 (30.3)	< 0.001
HbsAg (N,%)^e^	1 (0.6)	1 (1.2)	0	6 (12)	5 (20)	0	2 (7.4)	0	< 0.001

*DM* diabetes mellitus, *SAH* systemic arterial hypertension, *eGFR* estimated glomerular filtration rate), *LN* lupus nephritis, *DKD* diabetic kidney disease, *SV* systemic vasculitis, *S-FSGS* secondary focal segmental glomerulosclerosis, *S-MPGN* secondary membranoproliferative glomerulonephritis, TMA: thrombotic microangiopathy, *A-M-LCDD* amyloidosis, multiple myeloma, light chain deposition disease; Other diagnoses: other secondary glomerulopathies

^1^N(%): Chi-square test

^2^Mean ± standard deviation: ANOVA

^3^Median (interquartile range): Kruskal-Wallis test

Number of patients with measured serological parameters: ^a^C3: 455; ^b^C4: 455; ^c^anti-HIV: 461; ^d^anti-HCV: 465; ^e^HbsAg: 461

**Fig. 8:**
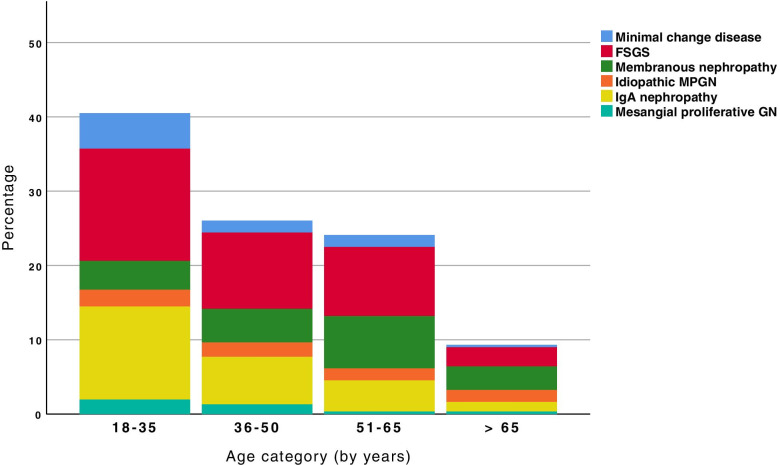
Frequency of primary glomerular diseases by age category. FSGS: focal segmental glomerulosclerosis, MPGN: membranoproliferative glomerulonephritis, GN: glomerulonephritis. Percentage values (%) according to each age category and relative to each disease: minimal change disease, FSGS, membranous nephropathy, idiopathic MPGN, IgA nephropathy, and mesangial proliferative GN: 18-35 years (*N* = 126): 11,9; 37,3; 9,5; 5,6; 31; 4,8. 36-50 years (*N* = 81): 6,2; 39,5; 17,3; 7,4; 24,7; 4,9. 51-65 years (*N* = 75): 6,7; 38,7; 29,3; 6,7; 17,3; 1,3. > 65 years (*N* = 29): 3,4; 27,6; 34,5; 17,2; 13,8; 3,4

**Fig. 9:**
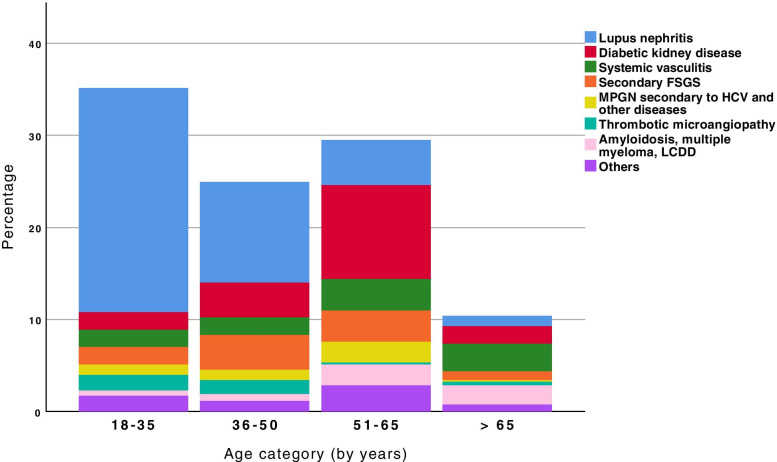
Frequency of secondary glomerular diseases by age category. FSGS: focal segmental glomerulosclerosis; MPGN: membranoproliferative glomerulonephritis; HCV: hepatitis C virus; LCDD: light chain deposition disease. Percentage values (%) according to each age category and relative to each disease, respectively lupus nephritis, diabetic kidney disease, systemic vasculitis, secondary FSGS, MPGN secondary to HCV and other diseases, thrombotic microangiopathy, amyloidosis, multiple myeloma, LCDD, and others: 18-35 years (*N* = 186): 69,4; 5,4; 5,4; 3,2; 4,8; 1,6; 4,8; 5,4. 36-50 years (*N* = 132): 43,9; 15,2; 7,6; 15,2; 4,5; 6,1; 3,0; 4,5. 51-65 years (*N* = 157): 16,7; 34,6; 11,5; 11,5; 7,7; 0,6; 7,7; 9,6. > 65 years (*N* = 55): 10,9; 18,2; 29,1; 9,1; 1,8; 3,6; 20; 7,3

Figures 8 and 9 show the distribution of primary and secondary glomerulopathies by age group. There was a significant difference in the percentages by age group, both in the primary (*p* = 0.012) and secondary forms of glomerulopathy (*p* < 0.001). Among the patients with primary glomerulonephritis, there was a higher prevalence of FSGS and IgAN for those between 18 and 35 years of age and those between 36 and 50 year of age. For patients 51-65 years of age, FSGS and MN had a higher prevalence, and there was a decrease in IgAN prevalence in this age group compared to the other age groups. The number of patients over 65 years of age who received a renal biopsy was lower (29%), and in this age group, FSGS and MN predominated. Among the patients with secondary glomerulopathies, LN was most prevalent in patients 18-35 and 36-50 years of age. In patients 51-65 years of age, DKD predominated; for patients over 65 years, systemic vasculitis and A-MM-LCDD were the prevalent pathologies, and there was an increase in the frequency of 2nd FSGS.

**Fig. 10:**
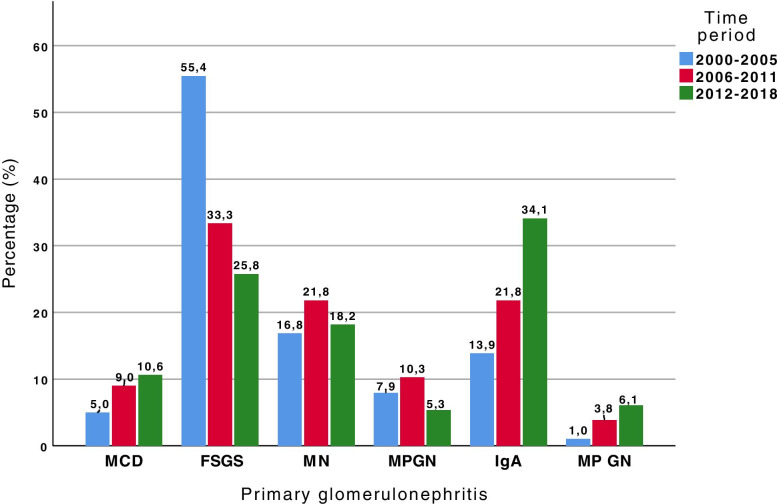
Prevalence of primary glomerular diseases over three time periods, from 2000 to 2005, 2006 to 2011 and 2012 to 2018. Comparing the causes of glomerulonephritis in each time period, statistical significance was found for FSGS (*p* < 0.001) and IgAN (*p* = 0.001); *p* > 0.05 for MCD, MN, MPGN and MesPGN. MCD: minimal change disease; FSGS: focal segmental glomerulosclerosis; MN: membranous nephropathy; MPGN: membranoproliferative glomerulonephritis; IgAN: IgA nephropathy; MesPGN: mesangial proliferative glomerulonephritis

**Fig. 11:**
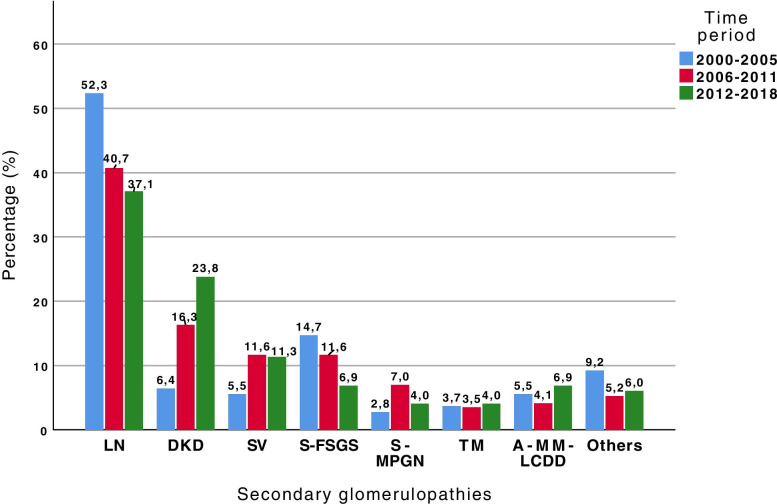
Prevalence of secondary glomerular diseases according to three time periods, from 2000 to 2005, 2006 to 2011 and 2012 to 2018. Comparing the causes of glomerulonephritis in each time period, statistical significance was found for LN (*p* = 0.027), DRD (*p* < 0.001), and a trend to S-FSGS (*p* = 0.053); *p* > 0.05 for SV, S-MPGN, TMA, A-MM-LCDD and other diagnoses. LN: lupus nephritis; DKD: diabetic kidney disease; SV: systemic vasculitis; S-FSGS: secondary focal segmental glomerulosclerosis; S-MPGN: secondary membranoproliferative glomerulonephritis; TMA: thrombotic microangiopathy; A-M-LCDD: amyloidosis, multiple myeloma, light chain deposition disease

The temporal variation of primary and secondary glomerulopathies in the three time periods showed a significant increase in the percentage of IgAN (*p* = 0.001) and a reduction in patients with FSGS (*p* < 0.001) over time, with no difference observed in the prevalence of MCD, MN, MPGN and MesPGN (*p* < 0.05) (Fig. 10). Among the secondary glomerulopathies, there was a significant reduction in LN prevalence (*p* = 0.027) and increase in DKD prevalence (*p* < 0.001) over time, and a tendency for 2nd FSGS incidence to decrease over time (*p* = 0.053) (Fig. 11). There was no difference in the variation of systemic vasculitis, 2nd MPGN, TMA, A-MM-LCDD or other pathologies over time.

Thirty-eight renal biopsies were sent for EM, of which four were non-representative. The representative biopsies were analysed, and the diagnosis was only changed from that obtained by optical microscopy and immunofluorescence in 10 (29%) patients. The most frequent diagnosis made by EM was FSGS with diffuse podocyte damage (44%), followed by Alport syndrome and thin basement membrane disease (12% each) and MCD and MN (6% each). There was one case of idiopathic nodular glomerulosclerosis and one case of C3 glomerulopathy (Dense Deposit Disease). Five of the biopsies (14%) sent for EM had no significant findings and were considered normal kidneys.

In the period from 2000 to 2018, the Nephrology and Radiology departments performed 538 and 513 renal biopsies, respectively. When comparing renal biopsies based on the performer, the total number of glomeruli observed by optical microscopy was significantly higher in biopsies done by nephrologists (median and interquartile range: 15[10-22]) than in those performed by radiologists (12[8-16]), *p* < 0.001. However, there was no difference in the total number of glomeruli observed by immunofluorescence (4[2-7] and 3[2-6], respectively; *p* = 0.320) or in the number of non-representative biopsies (*N* = 14 and *N* = 15, respectively; *p* = 0.568).

## Discussion

This study showed the distribution of demographic, clinical and laboratory data and histopathological diagnoses of 1051 patients who underwent renal biopsies from 2000 to 2018 at our centre. The histological diagnoses showed more than half of the patients had secondary glomerulopathies (52.4%), 29.6% had primary glomerulonephritis, and the remaining patients had other pathologies, tubulointerstitial disease or hereditary nephropathies.

Nephrotic syndrome was a prevalent clinical presentation and indicated renal biopsy mainly in primary forms but also in some secondary glomerulopathies. Studies have shown a higher frequency of nephrotic syndrome in different glomerulopathies, mainly MCD, FSGS and MN [2, 6, 12, 21, 22], which has also been described in systematic reviews [17], and is mainly a clinical presentation of primary glomerulopathies [16, 18]. For patients with IgAN, nephritic syndrome and proteinuria/haematuria prevailed, which is in agreement with the data presented in other studies [6, 18, 19, 21]. In secondary glomerulopathies, nephrotic syndrome and proteinuria/haematuria were prevalent in patients with LN, nephrotic syndrome in patients with DKD, nephrotic syndrome and proteinuria with loss of renal function in patients with 2nd FSGS and monoclonal gammopathies, and nephritic syndrome in patients with systemic vasculitis. These findings contradict those described in some other studies [6, 12, 14, 19, 23], which can be attributed to different clinical and laboratory criteria for performing renal biopsy between countries or states within a country, leading to substantial variability in the prevalence of primary and secondary glomerulopathies.

For primary glomerulonephritis, FSGS was the prevalent histology, followed by IgAN, MN, MCD and MPGN, with frequencies similar to those described in Brazilian registries [12–14, 24] and in North and South American countries [4, 5, 16, 25]. In the study by Polito et al. [12], the prevalence of FSGS, MN, IgAN, MCD and MPGN was 24.6, 20.7, 20.1, 15.5 and 4.2%, respectively, which was similar to that described by Malafronte et al. [13], who reported frequencies of 29.7, 20.7, 17.8, 9.1 and 7% for the same pathologies. Sim et al. [16] described a higher frequency of FSGS, followed by MN, MCD and IgAN, with an increase in FSGS and IgAN over 12 years in the follow-up period. Barrera-Herrera et al. [25] also reported a higher prevalence of FSGS and IgAN in the spectrum of primary glomerular diseases.

IgA nephropathy has a distinct epidemiological profile in different countries and continents. In Asia, its predominance is notorious and has been described in several studies [8, 9, 18, 19]. Yang et al. [8] reported a higher prevalence of IgAN followed by MPGN, MN and MCD, with FSGS described in only 4.6% of cases. In South Korea [9], the data are similar, with IgAN found in 28.3% of cases, and in Taiwan, Chiu et al. [18] also reported that IgAN had the highest prevalence (26%). In Japan [26], IgAN also predominated and clinically manifested as chronic nephritic syndrome. IgAN is also prevalent in some European countries, such as Italy [27], the Czech Republic [11] and Poland [10].

Lupus nephritis was the prevalent aetiology among the secondary glomerulopathies, as shown in Figs. 3 and 6, representing 41.1% of the cases, followed by DKD and systemic vasculitis. The analysis based on the histological class of LN revealed that classes III and IV (63%) and class V (17.8%) predominated, representing a total of 80.8% of cases. The prevalence of LN in our study did not differ from that in studies from other countries [5, 11, 19, 28]. In Brazil, Polito et al. [12] analysed 9617 renal biopsies, and LN was found in 42.3%, post-infectious glomerulonephritis in 20.4% and DKD in 10.1%. Malafonte et al. [13] reported LN in 66.2% of patients with secondary glomerulopathies, post-infectious glomerulonephritis in 12.5% and DKD in 6.2%. These data reflect the higher frequency of LN in several countries and in Brazil, showing similarity with our findings concerning the prevalence of secondary glomerulopathies.

Diabetic kidney disease was the second most frequent pathology among the secondary glomerulopathies. Usually, renal biopsy is indicated in patients with type 2 diabetes mellitus in the presence of atypical conditions to rule out a non-diabetic kidney disease, thus defining another pathology that potentially indicates immunosuppression. These criteria include abrupt nephrotic proteinuria, acute loss of renal function not compatible with the evolution of diabetes, short-term diabetes (less than 5 years), absence of diabetic retinopathy, and/or signs and symptoms of a systemic disease. Fiorentino et al. [29] conducted a pooled meta-analysis assessing 48 studies that analysed renal biopsies in patients with diabetes and reported that the prevalence of DKD, non-diabetic renal disease and mixed forms ranged from 6.5 to 94%, 3 to 82.9%, and 4 to 45.5%, respectively, overall. The most frequent non-diabetic nephropathy was IgAN, with a prevalence of 3-59%, followed by MN (7-35%), FSGS (17-37.7%) and ATIN (18-48.8%).

In a recent study, O’Shaughnessy et al. [5] evaluated data from 29 centres involving 42,603 patients and found a higher prevalence of FSGS and DKD in the USA/Canada. IgAN and FSGS predominated in Europe, IgAN and LN in Asia, and LN (38.1%) and FSGS (15.8%) in Latin America. Our results were similar to those obtained among Latin American patients in this study regarding LN (41.1%); however, the prevalence of FSGS was much higher in our population (37.3%). When taking the temporal variation that we found over the three periods analysed into account, the prevalence of DKD is consistent with the findings in the USA/Canada and the prevalence of IgAN is consistent with those described in the European and Asian continents.

Demographic data showed a predominance of Caucasians (85.5%) over those of African descent (14.1%), which was attributed to the ethnic profile of the southern region of Brazil. It is well known that there are differences in race in different regions of Brazil and worldwide. As an example, O’Shaughnessy et al. [5] reported the frequency of Caucasians and those of African descent in the USA/Canada (54.2 and 31.4%) and in Europe (98.2 and 0.8%), pointing to variations among countries on different continents. The number of centres involved in these studies was large, with 10 centres located in the USA/Canada and 14 centres located in Europe. However, data on ethnicity in Asia and Latin America were more restricted, with only 2 centres in Asia and 3 centres in Latin America, which limits comparisons to these regions.

The prevalence of each glomerulopathy varies by age group in different countries [6, 15, 18, 22]. Previous studies indicated that regarding primary glomerulopathies, the most frequent in the 18-50 year group were IgAN and FSGS, and FSGS and MN in the 51-65 year and > 65 year group. Regarding the secondary forms, LN was most prevalent between 18 and 35 years, DKD and 2nd FSGS between 36 and 60 years, and systemic vasculitis and monoclonal gammopathies in those over 65 years of age. These prevalences are consistent with those described in our study for all age groups together and for each age group analysed separately.

The temporal variation in glomerulopathies has been widely explored in many studies and has usually been assessed in the last four decades. Over three time periods, we observed a significant reduction in FSGS and an increase in IgAN among the primary glomerulopathies, a reduction in LN among the secondary glomerulopathies, an increase in DKD, and minor and non-significant variation in the other glomerulopathies over time. Studies have shown different temporal trends in both primary and secondary glomerulopathies. The data described by Sim et al. [16] between 2000 and 2011 showed an increase in the frequency of FSGS and IgAN over time and minor temporal variations with stabilization in pauci-immune vasculitis, MPGN, MCD and MN. In the study by Garau et al. [6], an increase in IgAN, MN, LN and DKD was observed between 1990 and 2014, with a decrease in FSGS observed after 2004. Brkovic et al. [30] compared 1987-2006 and 2007-2014 and reported an increase in MN and a reduction in non-IgA mesangioproliferative nephropathy over time, with no change in the frequency of other primary glomerulopathies. Dos-Santos et al. [31] showed an increase in the prevalence of FSGS between 1975 and 2006 and of LN and MN between 2006 and 2015, with a tendency for the prevalence of FSGS to decrease in this last period. It is likely that these temporal variations correlate with the heterogeneity of biopsy indications in different centres, changes in the population profile (for example, increasing age and ethnic variations), changes in lifestyle and socioeconomic diversity, all of which influence the epidemiological status of glomerulopathies and their variation over time.

The degree of chronicity in the histopathology also has significant prognostic value. Adjusting for demographic and laboratory data and clinicopathological diagnosis, Srivastava et al. [32] showed a higher risk for kidney disease progression in association with inflammation in the non-fibrous interstitium, moderate to severe tubular atrophy, interstitial fibrosis and global glomerulosclerosis, and arterial and arteriolar sclerosis. In our study, we did not perform this analysis based on the percentage of IFTA, global glomerulosclerosis or arteriolar damage described in the renal histology, which needs to be explored in a future study.

Our study has some limitations. As this is a retrospective study, the medical records of some patients showed incomplete demographic, clinical and laboratory data. As this was a single centre study, the results may not represent the actual prevalence of the studied disorders at other centres. In addition, our centre performs kidney biopsy for patients referred from other centres, limiting access to information in some cases, such as information regarding the clinical syndrome that indicated the biopsy and the patients’ clinical and laboratory data. However, of the 1051 patients included in this registry, the majority had accessible data in our centre’s medical records or data reported by physicians from external centres. In addition, all biopsy results were interpreted and reported by experienced nephropathologists, allowing for an aetiological diagnosis that defined the type of glomerulopathy or its non-representativeness.

## Conclusions

In this kidney biopsy registry, demographic and clinical data and the spectrum of renal histology of 1051 biopsies performed between 2000 and 2018 were described. FSGS and IgAN were the most common diagnoses in patients with primary glomerulonephritis, and LN and DKD were the most common diagnoses in patients with secondary glomerulopathies. Nephrotic syndrome was the major indication for biopsy, followed by proteinuria and proteinuria associated with haematuria. When comparing the temporal evolution of those glomerulopathies over three periods, there was a reduction in the frequency of FSGS and an increase in IgAN in the primary forms over time, and in secondary glomerulopathies, there was a reduction in LN with an increase in cases of DKD over time.

## Data Availability

All data generated or analysed during this study are included in this published article. The datasets used and/or analysed during the current study are available from the corresponding author on reasonable request.

## Abbreviations

CKD: Chronic kidney disease
IgAN: IgA nephropathy
FSGS: Focal segmental glomerulosclerosis
MCD: Minimal change disease
LN: Lupus nephritis
DKD: Diabetic kidney disease
MN: Membranous nephropathy
HCPA: Hospital de Clínicas de Porto Alegre
SAH: Systemic arterial hypertension
DM: Diabetes mellitus
eGFR: Estimated glomerular filtration rate
MDRD: Modification of Diet in Renal Disease
CKD-EPI: Chronic Kidney Disease-Epidemiology Collaboration
IPC: Proteinuria/creatininuria index
ANA: Anti-nuclear factor
ANCA: Anti-neutrophil cytoplasmic antibody
anti-GBM: Anti-glomerular basement membrane disease
MPGN: Membranoproliferative glomerulonephritis
MesPGN: Mesangial proliferative glomerulonephritis
2nd FSGS: Secondary FSGS
HIVAN: HIV-associated nephropathy
2nd MPGN: Secondary membranoproliferative glomerulonephritis
TMA: Thrombotic microangiopathy
A: Amyloidosis
MM: Multiple myeloma
LCDD: Light chain deposition disease
ATIN: Acute tubule-interstitial nephritis
CTIN: Chronic tubulo-interstitial nephritis
ATN: Acute tubular necrosis
TBMD: Thin basement membrane disease
HE: Haematoxylin and Eosin
PAS: Schiff’s Periodic Acid
PASM: Periodic Schiff-Methenamine
TM: Masson Trichrome
IFTA: Interstitial fibrosis and tubular atrophy
